# Chloroplast genome sequence and phylogenetic analysis of the hemiparasitic plant: *Taxillus sutchuenensis* var. *duclouxii* (Loranthaceae)

**DOI:** 10.1080/23802359.2026.2722799

**Published:** 2026-08-27

**Authors:** Jinju Zhao, Wei Liang, Rong Huang, Hui Wu, Yuqing Wei, Lei Zhang

**Affiliations:** ^a^ Key Laboratory of Ecological Protection of Agro-Pastoral Ecotones in the Yellow River Basin, National Ethnic Affairs Commission of the People’s Republic of China, School of Biological Science & Engineering, North Minzu University, Yinchuan, China; ^b^ Analysis and Testing Center of Ningxia Hui Autonomous Region, North Minzu University, Yinchuan, China

**Keywords:** Hemiparasitic plants, *Taxillus sutchuenensis* var. *duclouxii*, chloroplast genome, phylogenetic analysis

## Abstract

*Taxillus sutchuenensis* var. *duclouxii*, a shrub-like hemiparasitic plant with medicinal value. This study sequenced, assembled, and annotated the chloroplast genome of *T. sutchuenensis var. duclouxii* using Illumina high-throughput sequencing. The genome exhibits a typical circular quadripartite structure, with a total length of 122,537 bp and a GC content of 37.3%. It contains 104 genes, including 64 protein-coding genes, 32 tRNA genes, and 8 rRNA genes. Phylogenetic analysis shows that *T. sutchuenensis* var. *duclouxii* forms a clade with *T. nigrans* and *T. sutchuenensis*, indicating a close relationship. This study provides a basis for molecular identification and phylogenetic research in *Taxillus*.

## 1. Introduction

Chloroplasts, are essential organelles in plants, which serve as the primary site of photosynthesis and are involved in critical processes such as carbon fixation (Qin et al. 2023; Li et al. 2025). Chloroplast genomes (CPGs) range between 75 and 250 kb in length, with numerous copies per cell, and are generally maternally inherited in most plant species. CPGs have become valuable tools in comparative genomic and phylogenetic studies due to their structural simplicity, high conservation, and uniparental inheritance. They are increasingly being used to identify species, assess genetic and nucleotide diversity, resolve complex phylogenetic relationships, and infer evolutionary history (Quan et al. 2024). Consequently, CPGs have become essential markers in plant molecular systematics and conservation genetics, enabling researchers to efficiently track maternal lineages and evolutionary dynamics, thereby refining our understanding of plant biodiversity and adaptation mechanisms.

*Taxillus sutchuenensis* var. *duclouxii* (Lecomte) H. S. Kiu 1983 is a shrub-like hemiparasitic plant belonging to the genus *Taxillus* within the family Loranthaceae Juss. Qiu (1983) is distributed in northeastern Yunnan, Sichuan, Guizhou, western Hubei, and western Hunan, occurring in montane broad-leaved forests at elevations of 600–1600 meters. It parasitizes plants such as *Cyclobalanopsis* species, chestnut, pear tree, oil-tea camellia, tung oil tree, and members of the genus *Salix* (Qiu and Michael 2003). This plant exhibits therapeutic potential for conditions such as rheumatism, arthralgia, low back pain, and uterine disorders including bleeding and inadequate contractions (Qiu and Michael 2003). Despite the significant medicinal value of *T. sutchuenensis* var. *duclouxii*, research on the genetic foundation of this parasitic plant remains limited. In particular, studies investigating its chloroplast genome have yet to be reported.

Here, we assembled the chloroplast genome of *T. sutchuenensis* var. *duclouxii* for the first time and examined its general features. In addition, we investigated the phylogenetic position of *T. sutchuenensis* var. *duclouxii* within the genus *Taxillus*. This study aims to address two main questions: (1) What are the sequence characteristics of the chloroplast genome of *T. sutchuenensis* var. *duclouxii*? (2) What are the phylogenetic relationships among *Taxillus* species and the systematic position of *T. sutchuenensis* var. *duclouxii*?

## 2. Material and methods

**Figure 1.**
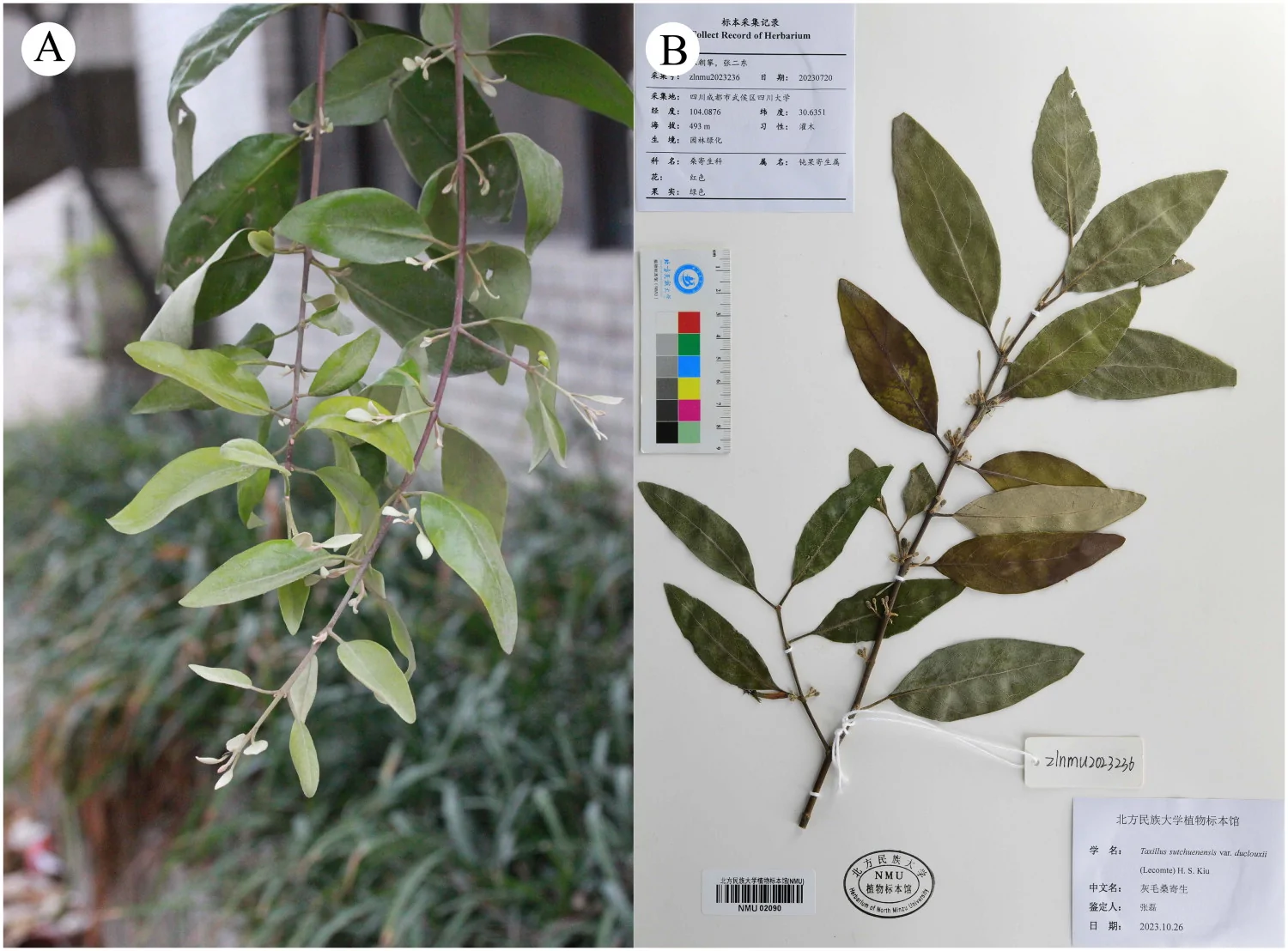
(A) An individual plant of *T. sutchuenensis* var. *duclouxii* taken from Sichuan University, Chengdu, Sichuan, China (photoed by Dr. Lei Zhang). (B) Herbarium of *T. sutchuenensis* var*. duclouxii* (young stem and leaf hairs grayish, leaf blade ovate to elliptic, abaxial surface grayish tomentose).

Healthy, fresh young leaves of *T. sutchuenensis* var. *duclouxii* were collected from the Sichuan University (Chengdu, Sichuan, China; coordinates: 104.0876° E, 30.6351° N) and dried in silica gel for DNA extraction. Flowering and fruiting branches were collected as voucher specimens, identified by Dr. Lei Zhang (zhangsanshi-0319@163.com), and deposited in the Herbarium of North Minzu University (https://www.cvh.ac.cn/ins/info.php?code=NMU, Lei Zhang: zhangsanshi-0319@163.com) under the voucher number zlnmu2023236 (Figure 1). Total genomic DNA was extracted using the hexadecyltrimethylammonium bromide (CTAB) method (Arseneau et al. 2017). Library with an average insert size of 350 bp were prepared using the NEBNext DNA Library Kit (New England Biolabs, Ipswich, MA, USA) and sequenced on an Illumina NovaSeq 6000 platform (San Diego, CA, USA), generating at least 6 Gb of 2 × 150 bp paired-end reads per sample. The sequencing depth coverage was conducted by Samtools (Li et al. 2009).

Low-quality reads (average quality score < 7 or containing > 10% ambiguous nucleotides) were removed, and the remaining high-quality data were assembled using NOVOPlasty v2.7.2 (Dierckxsens et al. 2017). Contigs were aligned to the reference chloroplast genome of *T. sutchuenensis* (KY996493) using Geneious v9.1.8 (Kearse et al. 2012) for gap filling and refinement. The complete chloroplast genome was annotated with Plann v1.1 (Huang and Cronk 2015), and circular map were produced using OGDRAW v1.2 (Lohse et al. 2013). Final annotated sequence was submitted to GenBank (Sayers et al. 2020).

For phylogenetic reconstruction, two datasets were constructed: a protein-coding gene (PCG) set and a whole plastome (WP) set. PCGs were extracted from the 20 CPGs (Table S1), aligned with PRANK v130410 (Löytynoja and Goldman 2008) based on amino acid translations, and concatenated into a supermatrix. For both datasets, we included *Macrosolen bibracteolatus* and *M. cochinchinensis* as outgroup. Phylogenetic analyses were performed for both datasets using maximum likelihood (ML) in RAxML v8.1.24 (Stamatakis 2014) with the GTR + Γ model and 1,000 bootstrap replicates, and Bayesian inference (BI) in MrBayes v3.2.6 (Ronquist et al. 2012) using the GTR+I + G model selected by jModeltest. The resulting phylogenetic trees were visualized using FigTree v1.4.2 (Rambaut 2018).

## 3. Results

**Figure 2.**
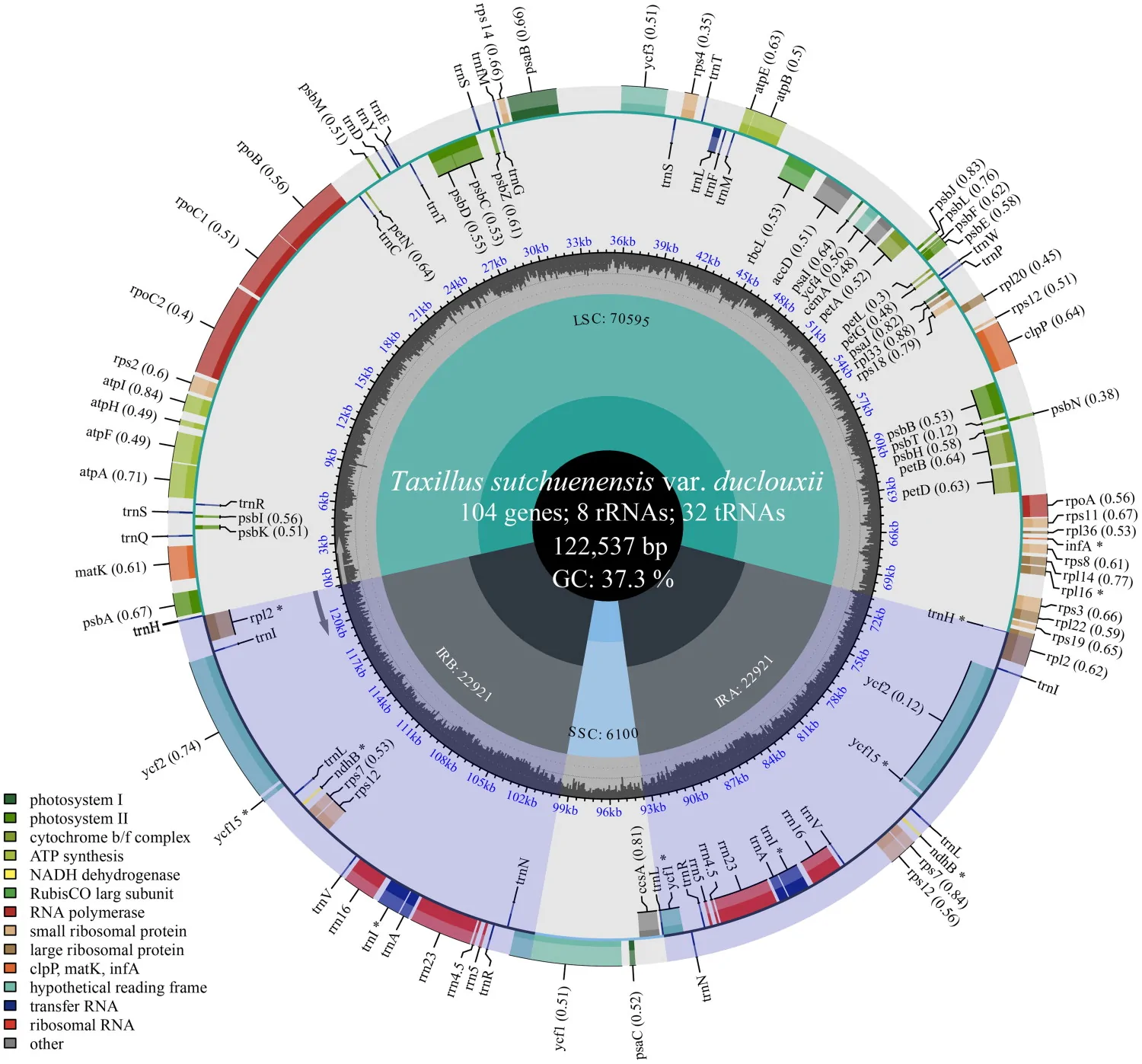
The chloroplast genome map of *T. sutchuenensis* var. *duclouxii* cp genome. Gene models including protein-coding genes, tRNA genes and rRNA genes are shown with various colored boxes in the outer track. The bold lines of the inner circle outline the extent of the inverted repeat regions (IRA and IRB), dividing the genome into small single-copy (SSC) and large single-copy (LSC) regions. Genes located on the inner and outer parts of the outer circle are transcribed in a clockwise and counterclock-wise direction, respectively. GC content (light gray) is shown in the inside track.

After quality control and preprocessing, we obtained at least six gigabases (Gb) of whole-genome sequencing data. The clean reads were used to assemble high-quality chloroplast genomes through a reference-guided approach. The total chloroplast genome of *T. sutchuenensis* var. *duclouxii* (PZ127291) was 122,537 bp long, with average depths of 7199×, 3605.97×, and 1387× for maximal, minimal, and average, respectively (Figure S1). It exhibited the typical quadripartite structure found in angiosperms, comprising a large single-copy (LSC) region of 70,595 bp, a small single-copy (SSC) region of 6100 bp, and two inverted repeats (IRa and IRb), each spanning 22,921 bp (Figure 2 and Figure S2). The *T. sutchuenensis* var. *duclouxii* cp genome encodes 104 predicted functional genes, which include 64 protein coding genes, 32 tRNA genes, eight rRNA genes, and two pseudogenes. Among these genes, nine protein-coding genes (*rpl*2, *rps*7, and *ycf*2), six tRNA genes (*trn*I-GAU, *trn*L-CAA, *trn*V-GAC, *trn*R-ACG, and *trn*N-GUU), and four rRNA genes (*rrn*4.5, *rrn*5, *rrn*16, and *rrn*23) were duplicated in the IR regions (Table S2). Additionally, the chloroplast genome contained 1 trans-splicing gene (Figure S3) and 7 cis-splicing genes (Figure S4). The GC content of the *T. sutchuenensis* var. *duclouxii* cpDNA was determined to be 37.3% overall. In the LSC, SSC, and IR regions, the GC content values were 34.7%, 26.4%, and 42.7%, respectively.

**Figure 3.**
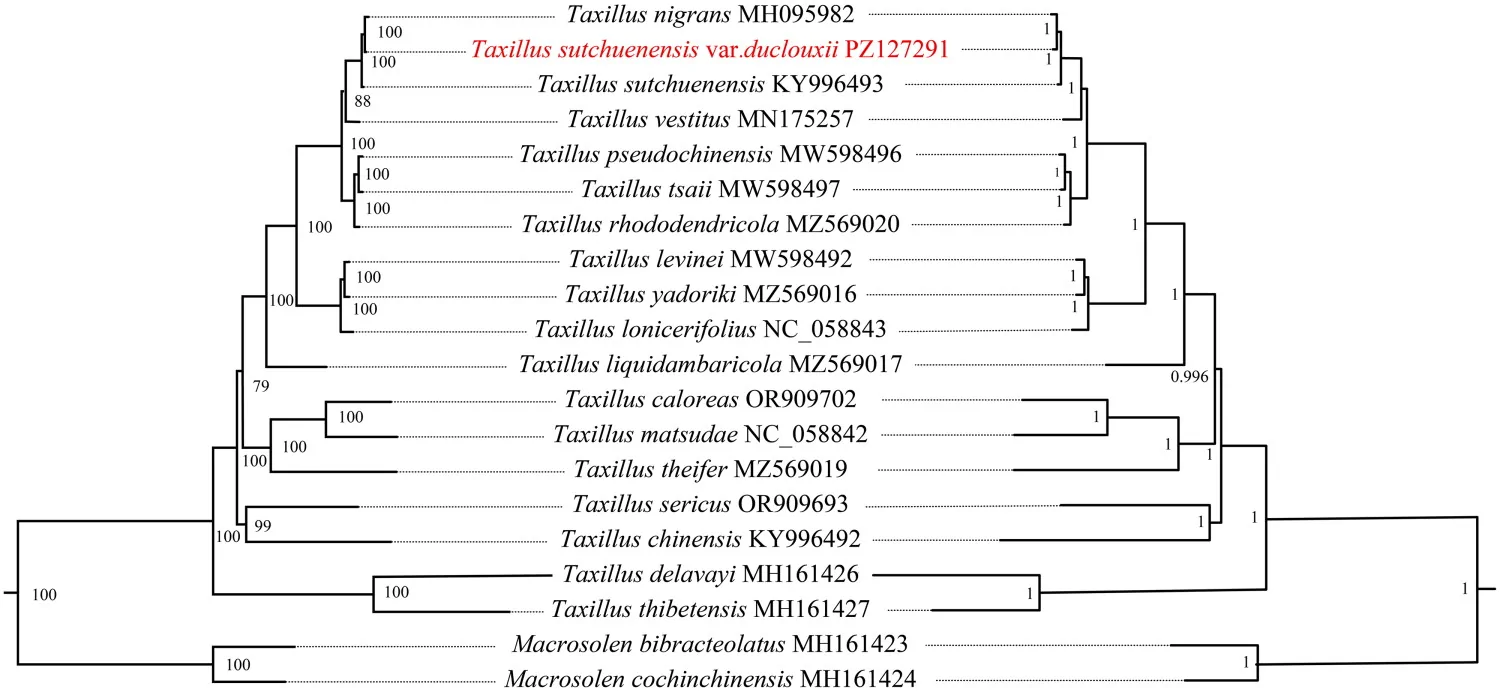
Phylogenetic tree based on whole chloroplast genome sequences for *taxillus* species with *macrosolen bibracteolatus* and *M. cochinchinensis* as the outgroup. The left and right trees are using the maximum likelihood (ML) and bayesian inference (BI) methods respectively. GenBank accession numbers: *Taxillus nigrans* MH095982 (Zhao et al. 2019), *Taxillus sutchuenensis* var. *duclouxii* (PZ127291), *Taxillus sutchuenensis* KY996493 (Li et al. 2017)*, Taxillus chinensis* KY996492 (Li et al. 2017), *Taxillus vestitus* MN175257 (Guo et al. 2026), *Taxillus pseudochinensis* MW598496 (Su et al. 2021), *Taxillus tsaii* MW598497 (Su et al. 2021), *Taxillus rhododendricola* MZ569020 (Su et al. 2021), *Taxillus levinei* MW598492 (Su et al. 2021), *Taxillus yadoriki* MZ569016 (Su et al. 2021), *Taxillus lonicerifolius* NC_058843 (Su et al. 2021), *Taxillus liquidambaricola* MZ569017 (Su et al. 2021), *Taxillus caloreas* OR909702 (Tang et al. 2024), *taxillus matsudae* NC_058842 (Su et al. 2021), *taxillus theifer* MZ569019 (Su et al. 2021), *Taxillus sericus* OR909693 (Tang et al. 2024), *Taxillus delavayi* MH161426 (Wu et al. 2023), *Taxillus thibetensis* MH161427 (Wu et al. 2023), *Macrosolen bibracteolatus* MH161423 (Nie et al. 2019), *Macrosolen cochinchinensis* MH161424 (Nie et al. 2019). The species from this study are shown in red on the phylogenetic tree.

We conducted phylogenetic analyses using complete chloroplast genomes from 18 *Taxillus* species, employing Maximum Likelihood (ML) and Bayesian inference (BI). The resulting trees exhibited congruent topologies with strong nodal support, consistently placing *T. sutchuenensis* var. *duclouxii* within a well-supported clade alongside two congeneric species, *T. nigrans* and *T. sutchuenensis* (Figure 3 and Figure S5). This robust phylogenetic reconstruction confirms the taxonomic placement of *T. sutchuenensis* var. *duclouxii* within the genus *Taxillus* (Loranthaceae).

## 4. Discussion and conclusion

Research on hemiparasitic plants is essential for understanding the evolutionary transition from autotrophic to parasitic organisms (Der and Nickrent 2008; Nickrent et al. 2010). The shift from autotrophy to parasitism imposes selective pressures on the evolution of chloroplast genomes, leading to reduced genome size, gene loss, and structural rearrangements (Neuhaus and Emes 2000; Wicke et al. 2016; Barrett et al. 2018). In this study, the chloroplast genome of *T. sutchuenensis* var. *duclouxii* was determined to be 122,537 bp in length and contained 104 genes, which is consistent with previous findings (Petersen et al. 2015; Shin and Lee 2018; Guo et al. 2021). For instance, the chloroplast genomes of *T. chinensis* and *T. delavayi* are 121,363 bp and 122,562 bp in length, each comprising 106 genes (Li et al. 2017). This study represents the first complete sequencing, assembly, and annotation of the chloroplast genome of *T. sutchuenensis* var. *duclouxii*, revealing the basic characteristics of the chloroplast genome of *T. sutchuenensis* var. *duclouxii* at the genetic level. These findings provide a crucial foundation for future investigations into the genetic structure and diversity of the genus *Taxillus*.

A phylogenetic tree constructed using complete chloroplast genome sequences from 18 *Taxillus* species, with the closely related two *Macrosolen* species as an outgroup, revealed that *T. sutchuenensis* var. *duclouxii, T. nigrans* and *T. sutchuenensis* formed a single clade with strong nodal support, indicating a robust taxonomic classification. Interestingly, within this clade, *T. sutchuenensis* var. *duclouxii* exhibits the closest relationship with *T. nigrans* rather than with the typical *T. sutchuenensis* variety. The above result might be due to ancient hybridization leading to chloroplast genome transfer, or by incomplete lineage sorting, rapid divergence causing retention of ancestral chloroplast variants. Our finding is consistent with previous phylogenetic analyses based on genomic data (Shin and Lee 2018; Jiang et al. 2023; Wang et al. 2023). Our findings further enrich the chloroplast genomic resources for the genus *Taxillus*. A thorough characterization of structural variation in the chloroplast genome of *T. sutchuenensis* var. *duclouxii* will require additional research, including population-level surveys and more comprehensive genome sequencing.

## Supplementary Material

Supplementary File.docx

## Data Availability

The *T. sutchuenensis* var. *duclouxii* chloroplast genome sequence data that support the findings of this study are openly available in NCBI (https://www.ncbi.nlm.nih.gov/) under the accession no. PZ127291. The associated BioProject, SRA and Bio-Sample numbers are PRJNA1441753, SRR37794313 and SAMN56667680, respectively.
