# Supplementary material for: Chloroplast genome sequence and phylogenetic analysis of the hemiparasitic plant: Taxillus sutchuenensis var. duclouxii (Loranthaceae)
Source: Mitochondrial DNA B Resour. 2026 Aug 27;11(9):1075–9. doi: 10.1080/23802359.2026.2722799 (PMC13523116; doi:10.1080/23802359.2026.2722799)
Supplement: Supplementary file 1 [file TMDN_A_2722799_SM4435.docx]

Chloroplast genome sequence and phylogenetic analysis of the hemiparasitic plant: *Taxillus sutchuenensis* var. *duclouxii* (Loranthaceae)

Jinju Zhao^1^, Wei Liang^1^, Rong Huang^2^, Hui Wu^1^, Yuqing Wei^1^, Lei Zhang^1*^

^1^ Key Laboratory of Ecological Protection of Agro-Pastoral Ecotones in the Yellow River Basin, National Ethnic Affairs Commission of the People’s Republic of China, School of Biological Science & Engineering, North Minzu University, Yinchuan 750021, China

^2^ Analysis and Testing Center of Ningxia HuiAutonomous Region, North Minzu University, Yinchuan 750021, China

* Corresponding author: Lei Zhang, zhangsanshi-0319@163.com

**ORCID**: Lei Zhang; https://orcid.org/0000-0001-5301-4658

**Table S1** Species information and data resources

| Species | Accession | References |
| --- | --- | --- |
| *Taxillus nigrans* | MH095982 | Zhao S, Li J, Ma R, Miao N, Mao Q, Mao K. 2019. Characterization of the complete chloroplast genome of *Taxillus nigrans*. Mitochondrial Dna Part B. 4(1):472-473. |
| *Taxillus sutchuenensis* | KY996493 | Li Y, Zhou JG, Chen XL, Cui YX, Xu ZC, Li YH, Yao H. 2017. Gene losses and partial deletion of small single-copy regions of the chloroplast genomes of two hemiparasitic *Taxillus* species. Scientific reports. 7(1):12834. |
| *Taxillus sutchuenensis* var. *duclouxii* | PZ127291 | The species from this study |
| *Taxillus chinensis* | KY996492 | Li Y, Zhou JG, Chen XL, Cui YX, Xu ZC, Li YH, Yao H. 2017. Gene losses and partial deletion of small single-copy regions of the chloroplast genomes of two hemiparasitic *Taxillus* species. Scientific reports. 7(1):12834. |
| *Taxillus vestitus* | MN175257 | Guo X, Ruan Z, Zhang G. 2019. The complete plastome of *Taxillus vestitus* (Loranthaceae), a hemiparasitic plant. Mitochondrial DNA Part B. 4(2):3188-3189. |
| *Taxillus pseudochinensis* | MW598496 | Su HJ, Liang Sl, Nickrent DL. 2021. Plastome variation and phylogeny of *Taxillus* (Loranthaceae). PLOS ONE. 16(8):e0256345. |
| *Taxillus tsaii* | MW598497 | Su HJ, Liang Sl, Nickrent DL. 2021. Plastome variation and phylogeny of *Taxillus* (Loranthaceae). PLOS ONE. 16(8):e0256345. |
| *Taxillus levinei* | MW598492 | Su HJ, Liang Sl, Nickrent DL. 2021. Plastome variation and phylogeny of *Taxillus* (Loranthaceae). PLOS ONE. 16(8):e0256345. |
| *Taxillus yadoriki* | MZ569016 | Su HJ, Liang Sl, Nickrent DL. 2021. Plastome variation and phylogeny of *Taxillus* (Loranthaceae). PLOS ONE. 16(8):e0256345. |
| *Taxillus liquidambaricola* | MZ569017 | Su HJ, Liang Sl, Nickrent DL. 2021. Plastome variation and phylogeny of *Taxillus* (Loranthaceae). PLOS ONE. 16(8):e0256345. |
| *Taxillus theifer* | MZ569019 | Su HJ, Liang Sl, Nickrent DL. 2021. Plastome variation and phylogeny of *Taxillus* (Loranthaceae). PLOS ONE. 16(8):e0256345. |
| *Taxillus rhododendricola* | MZ569020 | Su HJ, Liang Sl, Nickrent DL. 2021. Plastome variation and phylogeny of *Taxillus* (Loranthaceae). PLOS ONE. 16(8):e0256345. |
| *Taxillus caloreas* | OR909702 | Tang L, Wang T, Hou L, Zhang G, Deng M, Gu X. 2024. Comparative and phylogenetic analyses of loranthaceae plastomes provide insights into the evolutionary trajectories of plastome degradation in hemiparasitic plants. BMC Plant Biology. 24(1). |
| *Taxillus sericus* | OR909693 | Tang L, Wang T, Hou L, Zhang G, DengM, Gu X. 2024. Comparative and phylogenetic analyses of loranthaceae plastomes provide insights into the evolutionary trajectories of plastome degradation in hemiparasitic plants. BMC Plant Biology. 24(1). |
| *Taxillus matsudae* | NC_058842 | Su HJ, Liang Sl, Nickrent DL. 2021. Plastome variation and phylogeny of *Taxillus* (Loranthaceae). PLOS ONE. 16(8):e0256345. |
| *Taxillus lonicerifolius* | NC_058843 | Su HJ, Liang Sl, Nickrent DL. 2021. Plastome variation and phylogeny of *Taxillus* (Loranthaceae). PLOS ONE. 16(8):e0256345. |
| *Taxillus thibetensis* | MH161427 | Wu L, Fan P, Zhou J, Li Y, Xu Z, Lin Y, Yao H. 2023. Gene losses and homology of the chloroplast genomes of *Taxillus* and *Phacellaria* species. Genes. 14(4)：943. |
| *Taxillus delavayi* | MH161426 | Wu L, Fan P, Zhou J, Li Y, Xu Z, Lin Y, Yao H. 2023. Gene losses and homology of the chloroplast genomes of *Taxillus* and *Phacellaria* species. Genes. 14(4)：943. |
| *Macrosolen bibracteolatus* | MH161423 | Nie L, Cui Y, Wu L, Zhou J, Xu Z, Lin Y, Yao H. 2019. Gene losses and variations in chloroplast genome of parasitic plant macrosolen and phylogenetic relationships within santalales. International Journal of Molecular Sciences. 20(22):5812. |
| *Macrosolen cochinchinensis* | MH161424 | Nie L, Cui Y, Wu L, Zhou J, Xu Z, Lin Y, Yao H. 2019. Gene losses and variations in chloroplast genome of parasitic plant macrosolen and phylogenetic relationships within santalales. International Journal of Molecular Sciences. 20(22):5812. |

**Table S2** Functional classifcation of chloroplast genes of *Taxillus sutchuenensis* var. *duclouxii*

|  | Gene family | Gene name |
| --- | --- | --- |
| Photosynthesis related genes | Subunits of photosystem I(4) | *psa*B, C, I, J |
|  | Subunits of photosystem II(14) | *psb*A, B, C, D, E, F, I, J, K, L, M, N, T, Z |
|  | Subunits of ATP synthase(6) | *atp*A, B, E, F*, H, I |
|  | Subunits of cytochrome b/f complex(6) | *pet*A, B*, D*, G, L, N |
|  | Subunit of rubisco(1) | *rbc*L |
| Transcription and translationrelated genes | Large subunit of ribosome(7) | *rpl*14, 20, 22, 33, 36, 2* (×2) |
|  | Small subunit of ribosome(12) | *rps*2, 3, 4, 7 (×2), 8, 11, 14, 18, 19, 12** (×2) |
|  | DNA dependent RNA polymerase(4) | *rpo*A, B, C1*, C2 |
|  | RNA Ribosomal RNAs(8) | *rrn*4.5 (×2), 5 (×2), 16 (×2), 23 (×2) |
|  | RNATransfer RNAs(32) | *trn*H-GUG*, Q-UUG, S-GCU, R-UCU, C-GCA, D-GUC, Y-GUA, E-UUC, T-GGU, S-UGA, G-UCC, M-CAU, S-GGA, T-UGU, L-UAA*, F-GAA, M-CAU, W-CCA, P-UGG, I-CAU* (×2), L-CAA (×2), V-GAC (×2), A-UGC* (×2), R-ACG (×2), N-GUU (×2), L-UAG |
| Others genes | c-type cytochrom synthesis gene(1) | *ccs*A |
|  | Envelop membrane protein(1) | *cem*A |
|  | Maturase(1) | *mat*K |
|  | Protease(1) | *clp*P** |
|  | Subunit of Acetyl-CoA-carboxylase(1) | *acc*D |
| Unknown function genes | Conserved open reading frames(5) | *ycf*1, 2 (×2), 3**, 4 |

#* represents a gene with one intron,** represents a gene with two introns,×2 indicates two copies.

**Figure S1** Coverage depth distribution of the *T. sutchuenensis* var. *duclouxii*.

**Figure S2** Collinearity map of *T. sutchuenensis* var. *duclouxii*. Gene models including protein-coding genes, tRNA genes and rRNA genes are shown with various colored boxes in the outer track. The bold lines of the inner circle outline the extent of the inverted repeat regions (IRA and IRB), dividing the genome into small single-copy (SSC) and large single-copy (LSC) regions. Genes located on the inner and outer parts of the outer circle are transcribed in a clockwise and counterclock-wise direction, respectively. GC content (light gray) are shown in the inside track. Red and green lines indicate collinear blocks between different regions.

**Figure S3** Structure of trans-splicing genes in the *T. sutchuenensis* var. *duclouxii*.

**Figure S4** Structure of Cis-splicing genes in the *T. sutchuenensis* var. *duclouxii*.

**Figure S5** Phylogenetic tree based on 59 PCGs for the *Taxillus* species and outgroup.The left and right trees are use the maximum likelihood (ML) and bayesian inference (BI) methods respectively. The species from this study are shown in red on the phylogenetic tree.
